# Real-Time Paddle Stroke Classification and Wireless Monitoring in Open Water Using Wearable Inertial Nodes

**DOI:** 10.3390/s25175307

**Published:** 2025-08-26

**Authors:** Vladut-Alexandru Dobra, Ionut-Marian Dobra, Silviu Folea

**Affiliations:** Faculty of Automatic Control and Computer Science, Technical University of Cluj-Napoca, 400114 Cluj-Napoca, Romania; ionut.dobra@aut.utcluj.ro (I.-M.D.); silviu.folea@aut.utcluj.ro (S.F.)

**Keywords:** paddle stroke classification, wearable sensors, real-time feedback, ESP32, MPU6050, ESP-NOW, gesture recognition, dynamic time warping, open-water sensing, human motion analysis

## Abstract

This study presents a low-cost wearable system for monitoring and classifying paddle strokes in open-water environments. Building upon our previous work in controlled aquatic and dryland settings, the proposed system consists of ESP32-based embedded nodes equipped with MPU6050 accelerometer–gyroscope sensors. These nodes communicate via the ESP-NOW protocol in a master–slave architecture. With minimal hardware modifications, the system implements gesture classification using Dynamic Time Warping (DTW) to distinguish between left and right paddle strokes. The collected data, including stroke type, count, and motion similarity, are transmitted in real time to a local interface for visualization. Field experiments were conducted on a calm lake using a paddleboard, where users performed a series of alternating strokes. In addition to gesture recognition, the study includes empirical testing of ESP-NOW communication range in the open lake environment. The results demonstrate reliable wireless communication over distances exceeding 100 m with minimal packet loss, confirming the suitability of ESP-NOW for low-latency data transfer in open-water conditions. The system achieved over 80% accuracy in stroke classification and sustained more than 3 h of operational battery life. This approach demonstrates the feasibility of real-time, wearable-based motion tracking for water sports in natural environments, with potential applications in kayaking, rowing, and aquatic training systems.

## 1. Introduction

Stroke detection in open water is critically important for several reasons, especially in the context of athlete performance, safety, and technology development. Stroke detection in stand-up paddleboarding is a key area of interest for improving performance, safety, and the development of smart outdoor technologies. Unlike traditional paddling or flatwater sports, SUP (stand-up paddle) presents unique challenges and opportunities in stroke monitoring due to its open-water dynamics and full-body engagement [1,2,3].

A proper stroke technique directly affects speed, endurance, and energy conservation in SUP. Detecting and analyzing paddle strokes helps paddlers refine their technique and reduce muscle fatigue over long distances. Many paddlers unknowingly favor one side or have imbalanced stroke rhythms [4]. Stroke detection enables the identification and correction of these inefficiencies [5].

Sudden changes in stroke rhythm or halt may indicate fatigue or potential accidents (e.g., falling off the board). Automated detection can trigger alerts for self-rescue or external assistance. This use-case can be highly valuable for lifeguards at open-water recreational lakes. In scenarios with multiple SUP users, automatic stroke detection can alert lifeguards if a paddler has stopped rowing, allowing them to quickly assess whether the person has fallen off the board or is simply resting.

With accurate stroke data, SUP athletes and recreational paddlers can receive real-time feedback or post-session analytics, enhancing coaching and self-improvement.

Wind, currents, and waves can alter stroke patterns. Stroke detection systems must adapt to these variables while maintaining accuracy, pushing to advance machine learning and signal processing solutions.

Combining stroke data with GPS and environmental metrics (like wave height or wind speed) allows for context-aware insights that help paddlers adjust their technique to changing conditions.

As SUP grows in popularity for leisure and fitness, stroke detection can bring structured training and safety insights to non-competitive paddlers. During races or group tours, stroke monitoring can enhance safety coordination and ensure that participants stay within performance or health thresholds [6].

Current systems are limited by poor adaptation to SUP’s unique movement patterns, lack of open datasets, and difficulty handling real-world noise. Research is growing, but few systems are designed specifically for stand-up paddleboarding in the field. While numerous studies have focused on traditional rowing disciplines, particularly kayaking, these have primarily emphasized the analysis of athlete biomechanics specific to those sports [7,8,9].

This study proposes a more versatile approach, developing a movement monitoring system that can be easily adapted to various sporting activities, with the capability to transmit real-time data to a central system. The proposed system builds upon two previously published studies from this year. The first study [10] introduced a dryland swimming simulator designed to help athletes refine their technique before entering the pool. This system was tested indoors using Wi-Fi communication; the performance of the stroke identification algorithms was evaluated using Pearson’s Correlation Coefficient (PCC) and Dynamic Time Warping (DTW) across multiple subjects to assess adaptability. The second study [11] presented several improvements, culminating in a system that is capable of monitoring swimming movements in water, providing real-time feedback to the user via a vibration motor. Additionally, it transmits data wirelessly to a central unit, which displays live feedback through a web interface. In both systems, only one arm was monitored, which was equipped with three nodes that work in a master–slave architecture.

This study presents a novel approach to stroke identification and monitoring for stand-up paddleboarding (SUP) by extending and adapting methodologies introduced in two prior studies. The proposal is a generalized and activity-adaptive system. The system architecture and sensor configuration require minimal modification to transition from swimming to paddleboarding, demonstrating the flexibility and scalability of the motion analysis framework across different sports domains. The central contribution lies in adapting stroke detection algorithms and sensor placement to suit the biomechanical characteristics of SUP, while maintaining the integrity of real-time feedback and wireless data transmission. The modularity of the system allows it to be repurposed for various endurance water sports with minimal reconfiguration.

To enable low-cost, real-time stroke monitoring in open-water environments, the system leverages ESP-NOW, which is a low-power wireless communication protocol developed by Espressif [12,13]. Unlike conventional Wi-Fi or Bluetooth solutions, ESP-NOW enables direct device-to-device communication without requiring network infrastructure, which is particularly advantageous in remote or open lake conditions where connectivity is limited or unavailable. Field tests were conducted in an open lake environment, where sensor nodes mounted on the paddleboarder’s arms transmitted stroke data directly to a nearby receiver master node. The receiver was connected to a central processing unit that was capable of displaying live feedback via a lightweight web-based interface. This configuration demonstrates the feasibility of real-time stroke analysis in uncontrolled, outdoor conditions using an affordable and energy-efficient hardware setup.

Several commercial and academic systems have been developed for stroke detection and movement analysis in water sports, particularly in kayaking, stand-up paddling (SUP), and rowing. Systems such as Motionize (approx. USD 169.99) [14,15] and Vaaka Cadence Sensors (approx. USD 225.00) [16] offer real-time stroke rate monitoring and basic technique insights, with compatibility for both kayaking and rowing through ANT+/Bluetooth connectivity. Vaaka, in particular, provides a long battery life (300+ h) and ease of mounting on the paddle shaft, making it suitable for extended field training without frequent maintenance. Similarly, rowing analytics platforms such as the NK SpeedCoach SUP 2 (approx. USD 471.91) [17] use onboard sensors and 5 Hz GPS to provide in-depth biomechanical feedback, including metrics such as stroke rate, speed, distance per stroke, and energy expenditure. It is specifically tailored for stand-up paddleboarding and includes a sunlight-readable display, as well as app connectivity for post-session review.

More advanced kayaking systems include the Paddle Power Meter (approx. USD 750.00) [18], which integrates force sensors directly into the paddle shaft to deliver real-time power output, stroke efficiency, and symmetry data. The system is aimed at elite athletes and coaches seeking granular performance metrics. Likewise, the Paddle-Lab Motion Sensor [19], developed in collaboration with Nelo, provides detailed motion analysis including stroke angle, roll, and paddle entry/exit characteristics. It targets a similar high-performance training demographic, integrating closely with the Nelo Coach platform.

These commercial systems vary significantly in complexity, cost, and battery performance. Lower-cost sensors like Vaaka offer basic yet reliable cadence tracking over long durations, whereas high-end systems such as Power Meter and Paddle-Lab enable richer biomechanical insights suited to elite training contexts. As such, the selection of a device depends on the sport (kayaking, SUP, or rowing), the depth of feedback required, and the logistical constraints of field deployment (e.g., battery life, wireless range, or mounting compatibility).

However, these systems are often proprietary, sport-specific, and cost-prohibitive, limiting their adaptability to other disciplines such as stand-up paddleboarding. Moreover, many rely on commercial GPS or Bluetooth communication, which may introduce latency or connectivity issues in remote settings. The academic literature has addressed kayak stroke detection using inertial measurement units (IMUs), yet SUP-specific research remains limited.

Table 1 presents a comparative overview of the aforementioned commercial systems and devices, highlighting their key features, specifications, and suitability for rowing and paddling activities in open-water environments. SUP refers to Stand-Up Paddle, DPS denotes Distance Per Stroke, and AAA indicates the type of batteries used.

This paper addresses that gap by providing a low-cost (approximately USD 150–USD 180), adaptable, and real-time monitoring system for SUP arm movement, contributing to the broader field of motion analysis in water-based sports. In addition to modular stroke detection, the system also explores the use of Wi-Fi-based ESP-NOW communication for efficient, infrastructure-free sensor data transmission, enabling real-time monitoring even in remote open-water environments.

## 2. Materials and Methods

As outlined in the introduction, the core of the proposed solution builds upon the methodologies presented in the previously published swimming monitoring systems [10,11]. In the following sections, the system architecture will be described with an emphasis on the newly introduced features specific to stand-up paddleboarding. The components and methods reused from prior work will be briefly summarized to avoid redundancy and maintain focus on the novel contributions of this study.

### 2.1. Hardware Setup

The proposed system comprises a total of six sensor nodes. Each node consists of an ESP32 WeMos Lolin Lite microcontroller (Espressif Systems, Shanghai, China), a 3.7 V 500 mAh Li-Po battery, and an MPU6050 (TDK InvenSense, San Jose, CA, USA) inertial measurement unit (IMU). All electrical connections were soldered; hot glue is applied to insulate exposed pins and components, providing basic mechanical and environmental protection. The three components are assembled using Velcro, which also facilitates attachment to the user’s arms for ease of deployment during testing [20]. In the present study, newly developed sensing devices were employed, and no vibration motors were integrated into the six sensor nodes. Consequently, the system does not provide direct haptic feedback to the user for stroke correction. This design choice aligns with the primary objective of the study, which focuses on accurate motion acquisition and real-time data transmission in an open space environment, rather than on providing user feedback mechanisms.

The nodes were positioned at specific locations on the arms—the wrist, elbow, and shoulder—in order to monitor upper-limb motion during paddleboarding. Since this activity relies primarily on arm movements to propel the board through the water, the devices were positioned near the major arm joints to effectively capture motion dynamics.

An additional seventh node acts as the central controller and receiver, which is responsible for collecting data from all other nodes. This central unit consists of a single ESP32 WeMos Lolin Lite and can be powered either via USB when connected directly to a laptop or via a Li-Po battery when operated wirelessly. A smartphone can be used to control the system and visualize the collected data; in the latter case, a laptop is used for powering the seventh node and for running the monitoring interface.

Due to budget constraints, no dedicated waterproof enclosure was used. For future iterations, the integration of a 3D-printed housing and full waterproof encapsulation is planned to improve durability and safety in aquatic environments. The sensor nodes worn on the user’s arms during stand-up paddleboarding were found to be sufficiently waterproof to withstand accidental immersion. This was demonstrated during the first experimental trial, as described in Section 3, in which the user unintentionally fell into the lake, causing all nodes to be fully submerged. The protective sealing applied to the nodes, consisting of hot glue, proved effective, with all components functioning normally after submersion.

**Figure 1:**
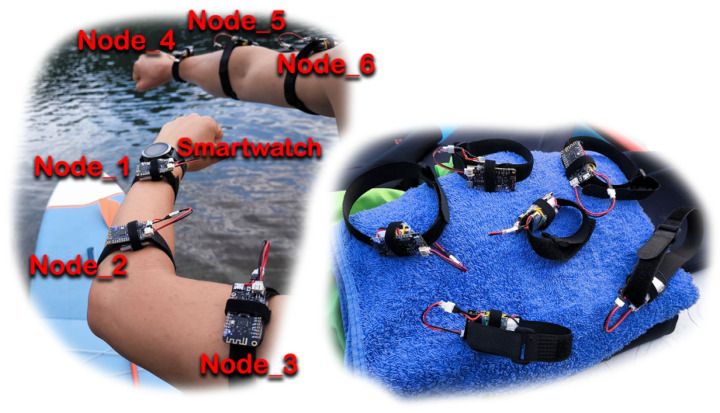
Placement of IMU nodes.

Figure 1 illustrates the hardware configuration, including the mounting of sensor nodes on both arms of the user.

An external device—a Garmin^®^ smartwatch [21] equipped with GPS functionality—was used during system monitoring, as illustrated in Figure 1. Its primary purpose was to verify the effective communication range between the IMU sensor nodes and the central control node, serving as a reference for distance estimation in open-water conditions. As will be presented in Section 3, a comparison is conducted between the stroke counts detected by the Garmin smartwatch and those recorded by the proposed system.

### 2.2. Communication and Arhitecture

**Figure 2:**
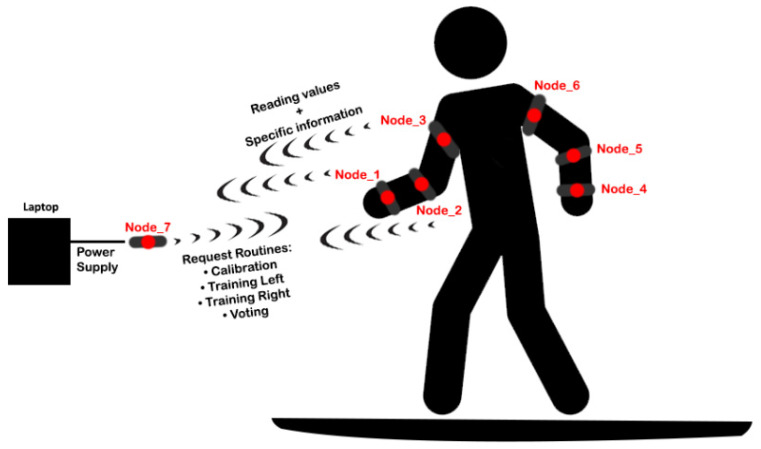
Communication diagram between devices.

The proposed system operates using Wi-Fi technology, specifically leveraging the ESP-NOW communication protocol for low-latency, infrastructure-free data transfer. Sensor nodes (Node 1 through Node 6) function as slave devices, while Node 7 serves as the central master node, which is responsible for coordinating data acquisition and communication with the user interface. Nodes 1 through 6 operate independently and do not communicate with each other. Figure 2 illustrates the communication between nodes.

The system is controlled via a web-based interface comprising four functional buttons and six real-time data display zones, one for each slave node. The interface allows the user to perform the following operations: (1) system calibration, (2) training of left-side paddle strokes, (3) training of right-side paddle strokes, and (4) continuous stroke monitoring.

The calibration function initializes the system by establishing a baseline using real-time data from the IMU sensors, specifically using accelerometer and gyroscope readings across the X, Y, and Z axes. The training functions for the left and right sides collect representative stroke patterns from the respective sides of the paddleboard. These patterns are processed to generate median reference values for each IMU axis, which are subsequently stored in the device’s non-volatile memory (NvM), ensuring data persistence across power cycles.

The stroke detection algorithm incorporates a voting mechanism that determines whether the sensor node is experiencing meaningful movement. When relevant motion is detected, the system records IMU data for a 2 s interval and forwards it to the stroke identification algorithm. In the absence of significant motion, data are discarded to conserve memory and processing resources. Furthermore, if no movement is detected, the system performs an automatic recalibration to account for sensor drift or environmental changes, maintaining the accuracy of subsequent measurements.

**Figure 3:**
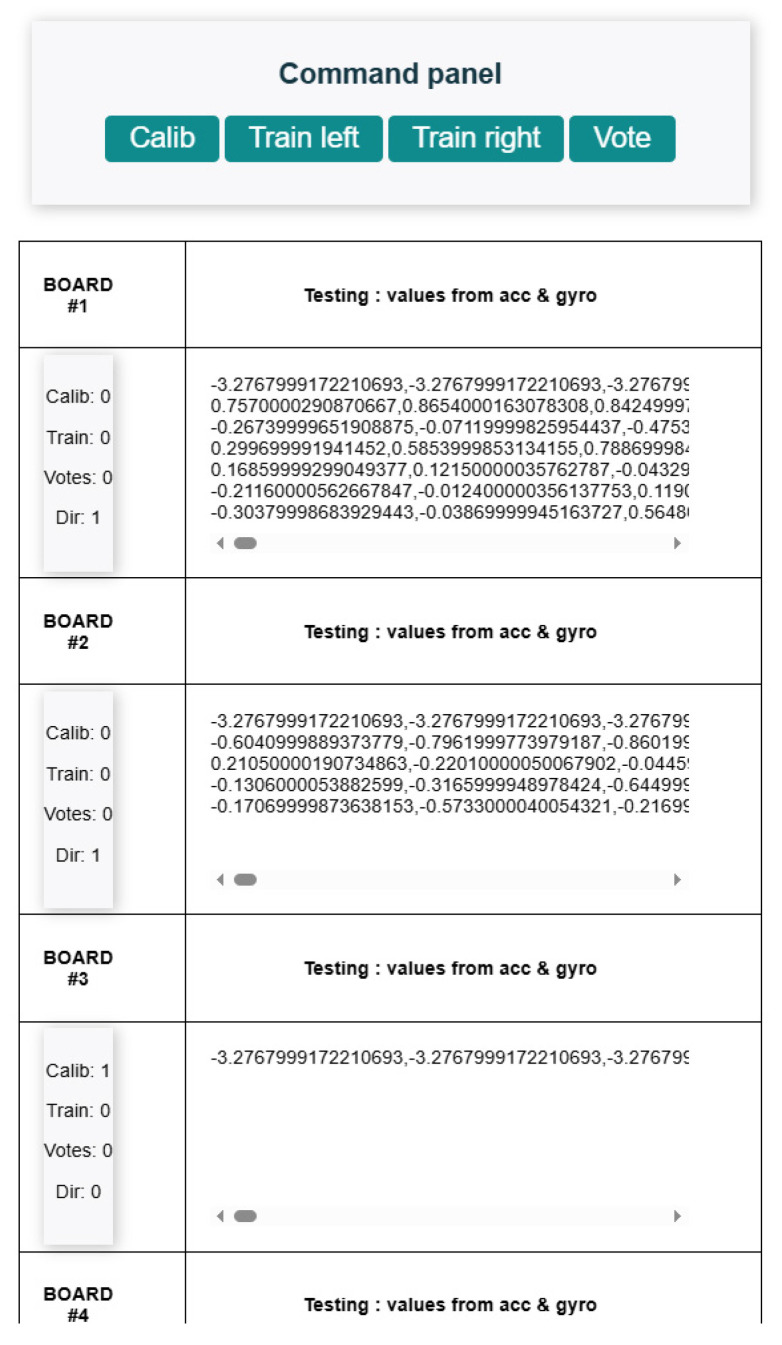
User interface for system control and data visualization.

Figure 3 illustrates the command panel and data visualization interface. Key improvements include the separation of training into two distinct phases—left and right stroke acquisition—and the addition of a ‘Dir’ field to indicate the detected direction of paddle movement. Furthermore, three additional data display panels have been incorporated, enabling the simultaneous monitoring of all six sensor nodes.

### 2.3. Stroke Detection Algorithm

The stroke detection algorithm is designed to distinguish between left and right paddle strokes in real time, based on previously trained motion patterns. To improve classification accuracy and simplify training, the routine is divided into two separate phases—one for left-side stroke acquisition and one for right-side stroke acquisition. This separation enables the system to more effectively learn and distinguish the characteristic motion profiles associated with each paddling direction.

Before stroke identification can occur, training data must be recorded. If training has been completed in a prior session, the resulting feature sets are stored in the microcontroller’s NvM, eliminating the need for retraining on subsequent uses.

For stroke recognition, the system employs the Dynamic Time Warping (DTW) algorithm to compare the incoming sensor data with the stored reference patterns. During the voting phase, each individual sensor node operates independently, continuously monitoring for significant movement. Upon detecting motion, the node captures a short time window of IMU data (accelerometer and gyroscope) and computes the DTW distances between the new data and both the left- and right-trained templates. The computing time is between 16 and 17 milliseconds.

The identification decision is based on the DTW cost values (denoted as $dtw_{total}$). The stroke is classified according to the pattern that yields the lower DTW cost, indicating a higher similarity between the live data and the trained reference. This enables real-time stroke detection and directional classification (left or right) with a lightweight yet robust computational approach.

Once a stroke is identified, the system transmits the determined direction, along with the computed similarity score, to the web interface, where the result is displayed in the ‘Dir’ field. The similarity score is expressed as a percentage ranging from 0 to 100%; it is derived from the cost value produced by the DTW algorithm.

Given that the user operates the system on an inflatable stand-up paddleboard in open-water conditions, where factors such as wind, wave disturbances, and the need for continuous balance introduce motion variability, the similarity function was adjusted to be less sensitive to minor fluctuations. Specifically, the scaling constant in the similarity function was modified to improve tolerance to environmental noise.

The similarity percentage is calculated using an exponential decay function of the form shown in Equation (1), where $dtw_{total}$ denotes the total DTW cost between the incoming stroke data and the trained template. To increase robustness, the constant originally set to 150,000 in previous work was reduced to 100,000, thereby lowering the sensitivity of the similarity function to small variations in movement patterns.

(1) $$Similarity\;percentage=100\times e^{-\left(\frac{dtw_{total}}{\frac{100,000}{dtw_{total}}}\right)\times dtw_{total}}$$

Each device stores a specific reference pattern obtained during the training phase. Training is performed with the device attached to a fixed position on the arm. If the device position is later modified during the voting routine, the resulting similarity scores for movement identification will decrease significantly due to misalignment with the trained pattern. The training and voting phases are presented in Supplementary Materials, Video S1.

In the case where a device is removed from the system, or if its battery is depleted, the corresponding node will no longer transmit data to the master device. This condition affects the system only by the absence of data from that specific node; the remaining nodes continue transmitting readings and computing similarity scores for each executed movement. For instance, if Node_2 (positioned on the left elbow) is removed, the system will lack information regarding elbow motion and therefore cannot assess whether the elbow contributes correctly to each stroke. However, the other slave nodes will still provide valid readings, allowing the evaluation of movement correctness based on the patterns established during training.

Each slave device acquires data continuously. Minor or insignificant movements are not considered eligible for similarity calculation; in such cases, the similarity score is set to zero. The algorithm differentiates between stationary states and motion. For light or short consecutive movements, the system may not acquire a complete dataset for evaluation. Specifically, each node records 40 sets of values for acceleration (ax, ay, az) and rotation (gx, gy, gz) during an eligible movement. If 10 consecutive readings indicate motion, the system acquires an additional 30 sets of values and subsequently evaluates the movement based on the full dataset. At the end of this process, a similarity percentage is generated. Motion detection is defined as variations exceeding 0.5 in either the accelerometer or gyroscope readings. Since the measurement range spans an interval of [−2; 2], corresponding to a total amplitude of 4 units, a deviation of 0.5 represents 12.5% of the full range. Movements surpassing this threshold are considered sufficiently significant to be evaluated by the system.

When the device strap is kept sufficiently tight and the sensor remains in its trained position, no significant deviations in similarity scores are observed. However, if the strap is loose, the sensor may shift on the user’s arm, producing readings from positions that differ from those used during training. This displacement introduces deviations, resulting in lower similarity percentages.

## 3. Results

**Figure 4:**
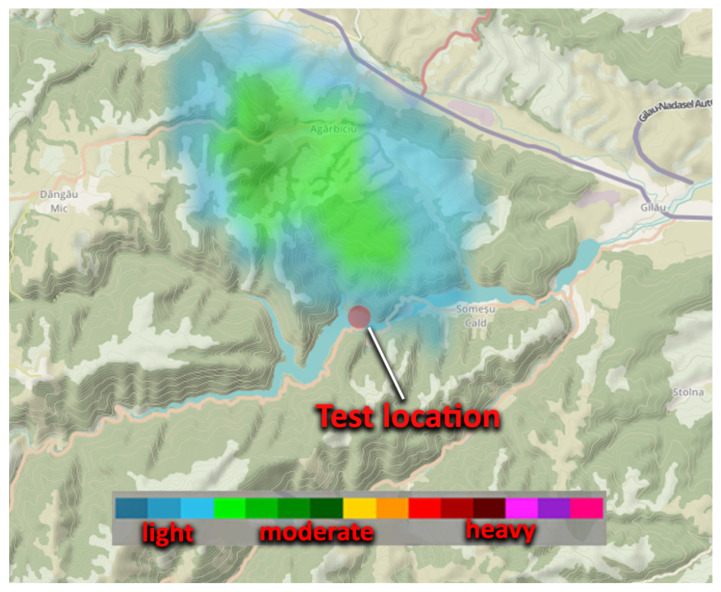
Radar for meteorological conditions.

The test environment was located in Cluj County, România, at Lake Tarnița. The meteorological conditions were favorable, with almost no wind and only a brief 10 min rain event, after which clouds moved away from the area and the lake remained calm. Figure 4 presents an image from the National Meteorology Association of Romania [22], illustrating the moment when the light rainfall passed over the test location at Lake Tarnița.

**Figure 5:**
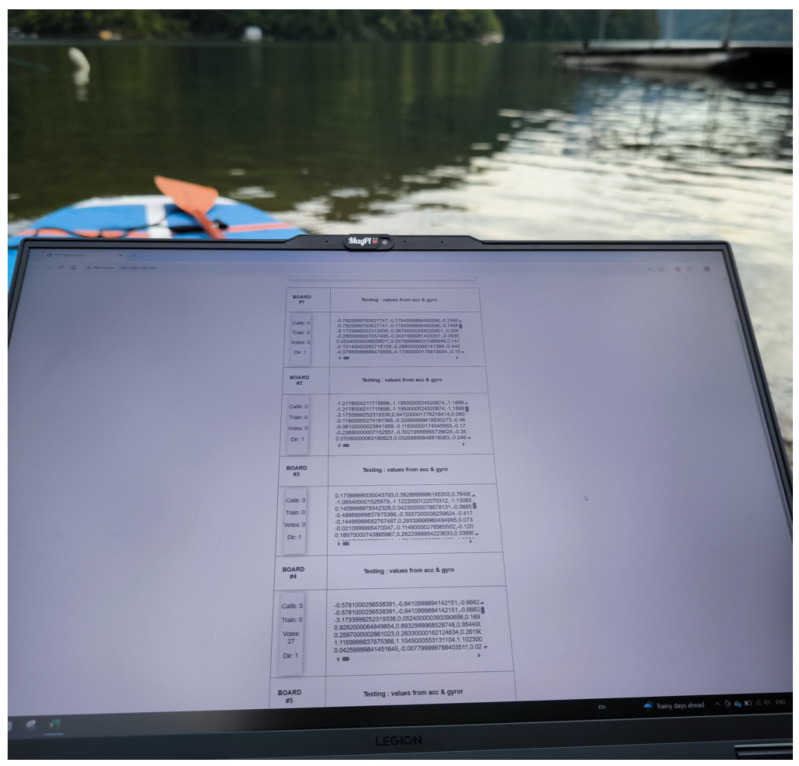
Lakeshore configuration for data visualization.

Two participants were involved in the testing phase. One remained on the lakeshore, where a laptop was used to visualize the data received from the slave nodes. The master node, which collected the values transmitted by the slave nodes, was connected to the laptop via USB (relevant exclusively to the device’s power supply). The laptop only accesses the webserver created by the master node. A web interface generated by the master node is hosted on a local server provided through the laptop’s Wi-Fi network. This process runs in parallel with data transmission between the slave nodes and the master node. The laptop creates a hotspot to which the master node connects. Within this network, the master node hosts a web server that can be accessed to control and visualize the system. Figure 5 illustrates the lakeshore-based data monitoring station.

The participant stationed at the lakeshore was responsible for the following tasks during the experiment:

- Controlling the system and activating the necessary routines, including calibration, left-side training, right-side training, and voting.
- Monitoring data transmission following each performed stroke.
- Visually counting the number of strokes performed for verification purposes.
- Checking for the end of data reception from the slave nodes.

As the lake was not crowded, verbal communication between the lakeshore observer and the paddleboard user was efficient.

The other participant in the experiment had the following responsibilities:

- Maintaining a standing position on the paddleboard and ensuring balance throughout the test.
- Performing the required actions for system initialization, including calibration, left-side training, and right-side training.
- Executing multiple strokes during the voting phase to facilitate data collection.
- Moving away from the shore to evaluate the Wi-Fi communication range.

In the following three experiments, the two participants are referred to as Subject A and Subject B.

**Figure 6:**
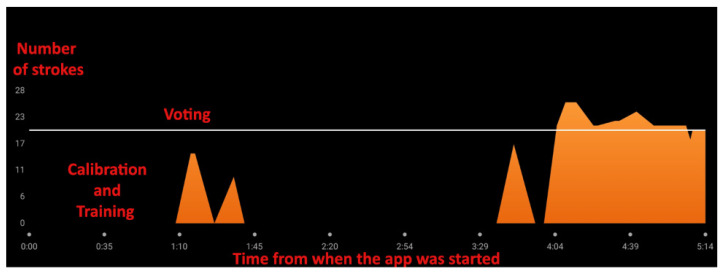
Smartwatch stroke count chart.

In addition to the proposed system, a Garmin smartwatch was utilized to track the path on the lake. The smartwatch’s SUP application was used to collect various data after the session, including GPS track, stroke count, heart rate monitoring, distance, and air temperature. To ensure consistent data acquisition and allow the observer stationed on the lakeshore sufficient time to visually monitor, count, and interpret each stroke, the paddle strokes were deliberately performed at a slower pace. However, under these conditions, the smartwatch results were inconclusive. As illustrated in Figure 6, generated with the Garmin mobile application, while the stroke-counting routine (voting) was initiated for the proposed system, the smartwatch failed to register any strokes between minute 1:45 and 3:29, despite continuous paddling activity. The graph of strokes identified by the smartwatch is shown in orange.

**Figure 7:**
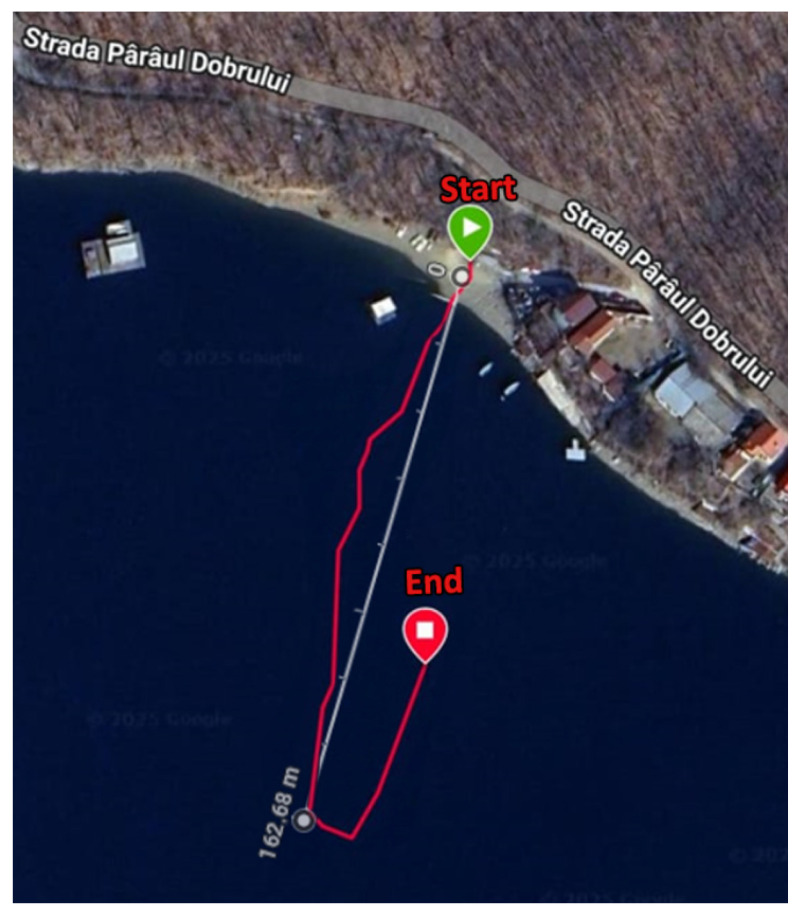
Path of the first test using a smartwatch for GPS monitorization.

After minute 3:30, an increase in detected strokes was observed, corresponding to the user performing more fluent and consistent paddle strokes following a turnaround maneuver. As shown in Figure 7, upon reaching a distance of 162 m from the starting point, the user executed a turn, after which the strokes became more regular. The smartwatch activity tracking application was manually stopped at the location marked by the red pin. The smartwatch recorded a total of 52 strokes.

Figure 7 illustrates the first test, where the red line represents the path recorded by the smartwatch and the white line corresponds to the distance measured using the Google Maps distance measurement feature.

During this test, paddle strokes were alternated in the voting phase by Subject A. The strokes were performed slowly to ensure that each stroke was accurately captured and monitored from the lakeshore by Subject B. A total of 47 strokes were executed until the furthest point, as indicated in Figure 7, was reached, corresponding to a distance of 162.68 m. This was verified by the external observer stationed on the lakeshore. Data loss began at approximately 100 m, based on observations from the web interface, where some messages transmitted from the slave nodes were not successfully received by the master node. Although communication was still partially functional at the maximum distance, not all transmitted messages were displayed on the webpage.

The second test focused primarily on stroke technique. Subject A was performing on the paddle board and Subject B was monitoring from the lakeshore. Since the training strokes were performed directly on water while standing on the paddleboard, the resulting stored patterns were well-suited for classification during the voting phase. Starting from the calibration position—with the paddle held above the water in front of the user—each stroke was executed slowly, with a duration of approximately 1.5 to 2 s per full motion.

After each stroke, the slave nodes computed Dynamic Time Warping (DTW) results by comparing the new input data with the pre-recorded patterns for left and right strokes. Based on the lower DTW cost, the system calculated a similarity percentage and determined the stroke direction. This information, along with the raw data, was then transmitted to the master node.

Each slave node required approximately 16–17 ms to compute the DTW results for a new data sequence during the voting phase. The data frame was then transmitted to the master node within the following 10 ms, resulting in a total delay of under 30 ms for processing and transmission.

The theoretical transmission time between a slave node and the master node using ESP-NOW is approximately 2–3 ms one-way at a data rate of 1 Mbps for 250-byte packets [23]. Upon arrival at the master node, two consecutive 10-ms processing tasks were used to receive and parse the incoming values. The data were then displayed on the laptop’s web interface within an additional 20–30 ms. In total, approximately 85 ms elapsed from the end of a stroke to the moment the corresponding data appeared on the web interface [24].

The ESP-NOW protocol employs Carrier Sense Multiple Access with Collision Avoidance (CSMA/CA). When multiple slave devices initiate transmission concurrently, each device first performs channel sensing; if the channel is occupied, transmission is postponed until it becomes available, with a randomly assigned backoff delay on the scale of microseconds. The master device stores incoming packets in a First-In–First-Out (FIFO) buffer, processing them in order of arrival and passing each sequentially to the OnDataRecv callback. Under typical conditions, airtime delays are approximately 1–2 ms per packet, with transmissions from six slaves in rapid succession requiring roughly 6–15 ms in total. In cases where collisions occur and retransmissions are required, each retry adds approximately 1–5 ms [24,25]. Although large data packets share the same communication channel and can generate busy traffic, this does not adversely affect system performance under the designed conditions. Even during concurrent transmissions, total delays remain small, on the order of 100–150 ms for all packets to be received [26].

**Figure 8:**
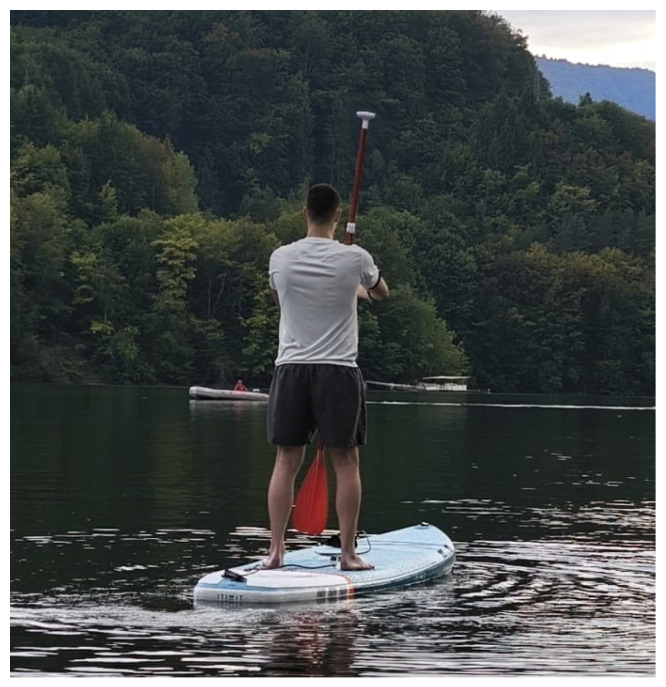
User position during system calibration.

Figure 8 illustrates the calibration position used during the experimental procedure.

**Figure 9:**
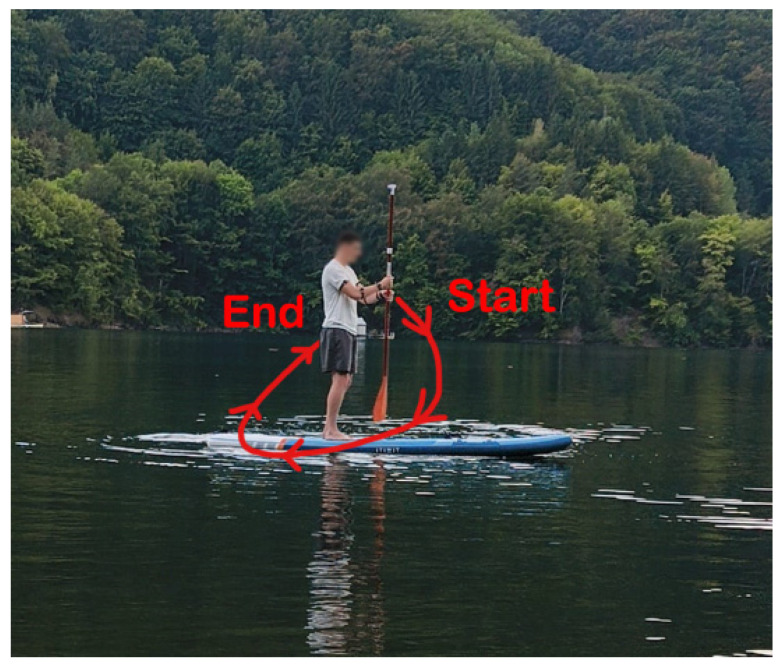
Left stroke motion execution scenario.

The training phase corresponds to the creation of the reference pattern, which serves as the basis for subsequent classification. It is essential that this phase is performed with high accuracy and without ambiguity, as the voting phase relies directly on the integrity of the trained pattern. Properly executed training ensures that the voting phase operates with greater efficiency and reliability. In Figure 9, left stroke movement is indicated.

**Figure 10:**
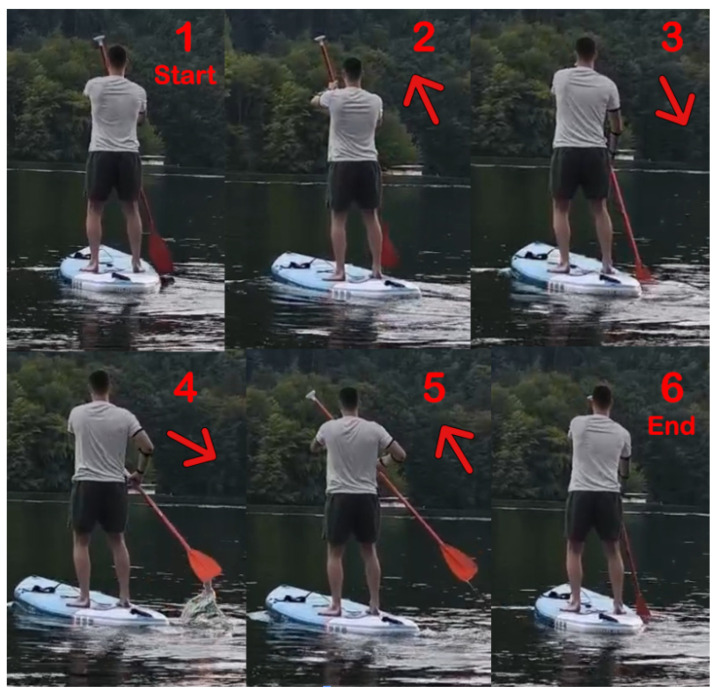
User position during training routine.

Figure 10 illustrates the training movements performed to record the reference pattern for the right-arm stroke. The arrows indicate the paddle movement.

**Figure 11:**
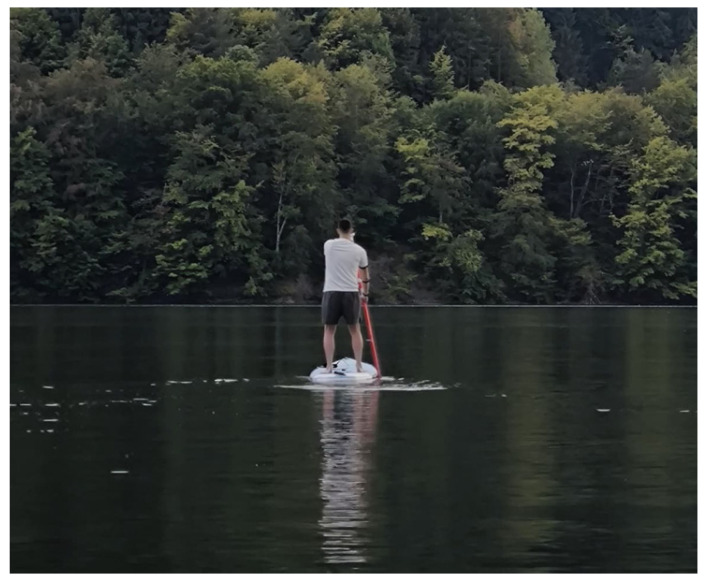
User position during voting routine.

Figure 11 illustrates the testing of the voting routine. The image captures the position of the paddle, the paddleboard, and the user’s stance during the execution of the stroke.

**Figure 12:**
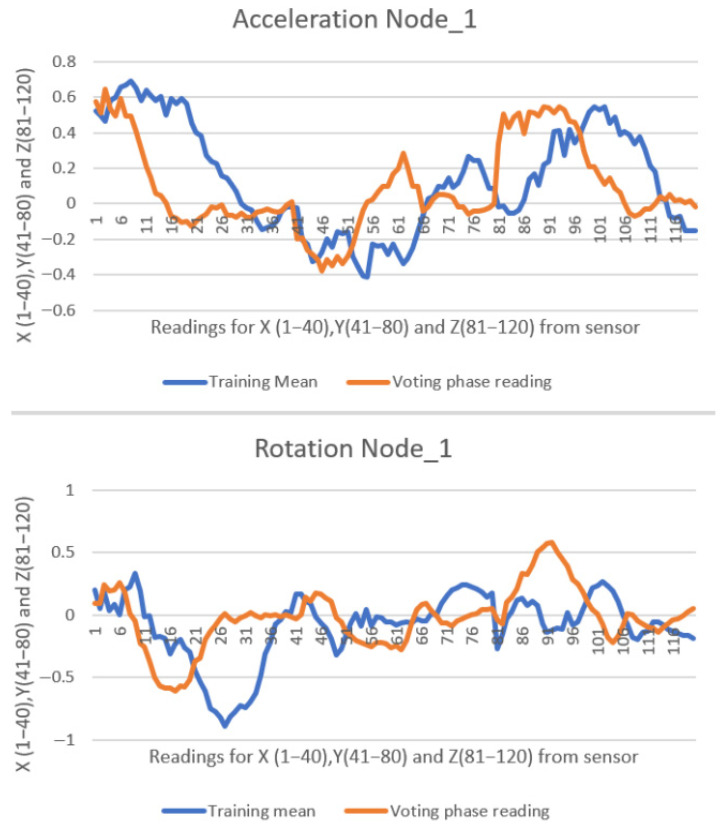
**Eighty-nine percent** similarity on Node_1 performing a left stroke.

**Figure 13:**
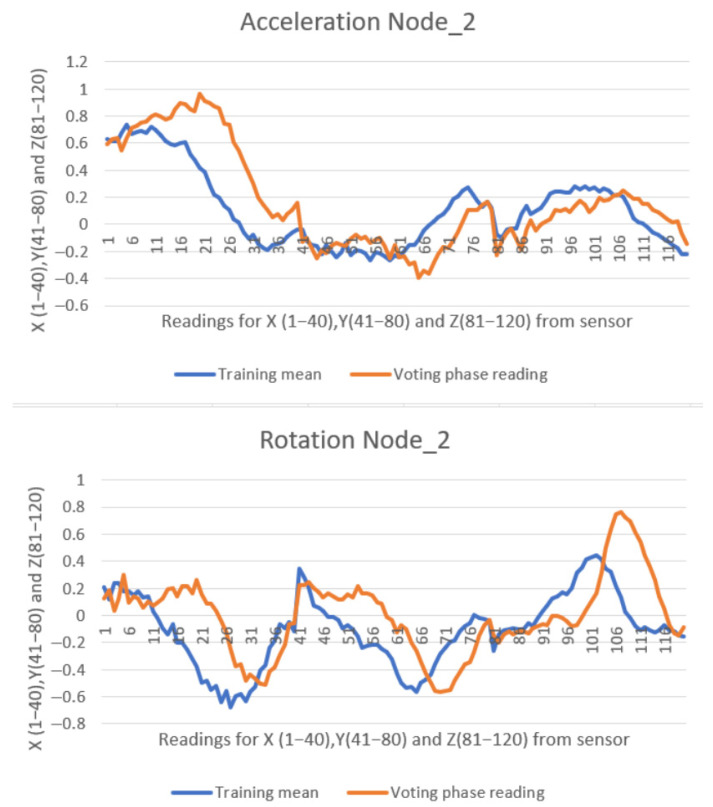
**Ninety-six percent** similarity on Node_2 performing a right stroke.

Each message received by the master device from a slave node contains 120 float-type values ranging from −2 to 2 (with six decimal places), along with two additional values indicating the stroke classification—vote or direction. In the second experiment, a total of 405 messages were transmitted from all six slave nodes to the master node over a 5 min duration, resulting in 49,410 data values being received and displayed in the web interface. All collected data were exported to Microsoft Excel Proffesional Plus 2021 for further analysis and visual inspection through plotted graphs. Due to the high volume of data, only two representative comparisons are illustrated in Figure 12 and Figure 13.

In comparison with the previous two studies [10,11], the graphical representations below exhibit a more pronounced misalignment between the recorded data (orange line) and the reference pattern (blue line). Despite this, the similarity scores remain high. This is attributable to modifications made to the similarity formula (Equation (1)), which were introduced to account for environmental noise factors present in the lake setting—such as paddleboard motion, user balance adjustments, minor wave activity, and light wind—during the testing sessions. The values are presented consistently, with 40 samples recorded for each of the X, Y, and Z axes of both the accelerometer and gyroscope. This results in a total of 120 data points being represented in the graph.

Figure 12 presents data from Node 1, showing the detection of a left stroke with 89% similarity to the trained pattern.

Figure 13 shows a right stroke detected by Node 2, with a 96% similarity score relative to its trained model.

In Table 2, the stroke identification output (‘Dir’) is listed. By calculating the mean identification percentage across all nodes, the system achieved an 84.40% average detection rate. This highlights the effectiveness of the distributed sensing setup, while also indicating potential areas for improvement in nodes with lower identification rates.

Nodes 1–3 were positioned on the left arm, while Nodes 4–6 were positioned on the right arm. In this experiment, Subject A was right-handed, and the results demonstrate a higher consistency in stroke identification on the right arm, with identification scores ranging between 83% and 87%. In contrast, Nodes 1–3 on the left arm exhibited greater variability, reflecting larger deviations in stroke classification. This inconsistency is attributed to the subject’s lower coordination in the non-dominant (left) arm. Furthermore, Node 3, which is positioned on the left shoulder, showed a particularly reduced stroke identification performance, further highlighting the impact of reduced left-arm coordination.

**Figure 14:**
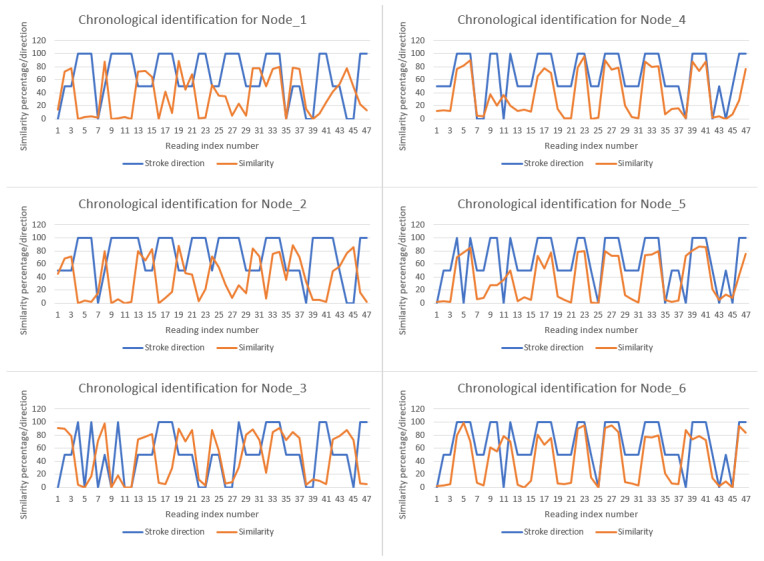
Stroke direction and similarity percentage across all nodes in consecutive readings.

To synchronize the output of the entire system based on movement identification, Nodes 1, 2, and 3 are primarily responsible for detecting left-hand strokes, as they are positioned on the left-hand side. In contrast, Nodes 3, 4, and 5 are primarily responsible for detecting right-hand strokes, being positioned on the right-hand side. The measurement values and computed similarity scores are transmitted to the master node (Node 7), where they are displayed in chronological order. Figure 14 illustrates how the system identifies each stroke sequentially. Each slave device reads data from the sensor, computes the Dynamic Time Warping (DTW) cost with the left-stroke reference pattern, and then computes the DTW cost with the right-stroke reference pattern. The smaller of the two DTW costs determines whether the stroke is classified as left or right. In the presented case, 47 strokes were recorded on the stand-up paddle (SUP). For each node, a graph displays the classification output (blue line: 50 = left stroke, 100 = right stroke, 0 = undetected stroke) along with the corresponding similarity score for that stroke (orange line).

The misclassified strokes, shown in Figure 14 as blue line segments with a value of 0, result from suboptimal stroke execution, poor balance on the paddleboard, and excessively rapid stroke movements. Since the system is designed for stroke learning and monitoring rather than high-speed use, the computed DTW costs for both patterns are comparatively small, reducing classification accuracy. Future improvements could address these limitations by resolving corner-case issues, increasing the volume and diversity of test data and refining the reference patterns through targeted training. Such enhancements would improve the system’s ability to reliably determine at least the stroke’s direction and position.

As the stand-up paddleboard moves farther from the lakeshore, some of the transmitted messages from the slave nodes are not successfully received by the master node. Table 2 summarizes the information collected during the voting routine, including all stroke identifications received from each node.

A current limitation in the implementation is the lack of synchronization between messages from different nodes, which restricts the ability to establish a coherent, time-aligned overall stroke pattern, as the smartwatch has (Figure 6). This issue could be addressed by including an additional field in each data packet to represent a timestamp. With such synchronization, temporal correlation and multi-sensor fusion would become feasible for a more robust pattern recognition.

However, the system architecture was adapted from a previously developed solution for swimming activity recognition, where real-time feedback was prioritized. In that context, each node delivered immediate feedback to the athlete via a vibration motor, and the focus was not on centralized data collection or web-based visualization. Consequently, timestamp synchronization was not originally considered essential in the system’s design.

**Figure 15:**
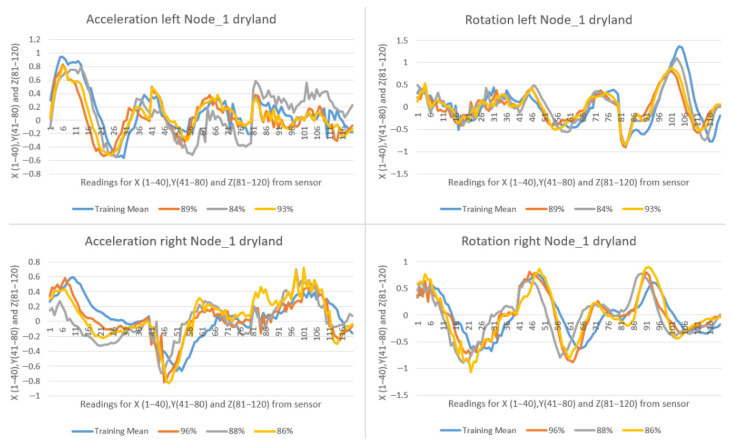
Indoor testing with high-similarity percentage strokes.

The results obtained from this test can be directly compared with those recorded in indoor conditions. During the indoor testing phase, stroke movements were performed to evaluate the accuracy of the system prior to testing in open-water conditions. Figure 15 presents the results from Node_1, showing multiple left and right stroke executions (orange, gray, and yellow lines) in comparison with the corresponding training pattern (blue line). The percentages shown in the figures represent the similarity scores, which are computed using both accelerometer and gyroscope data.

**Figure 16:**
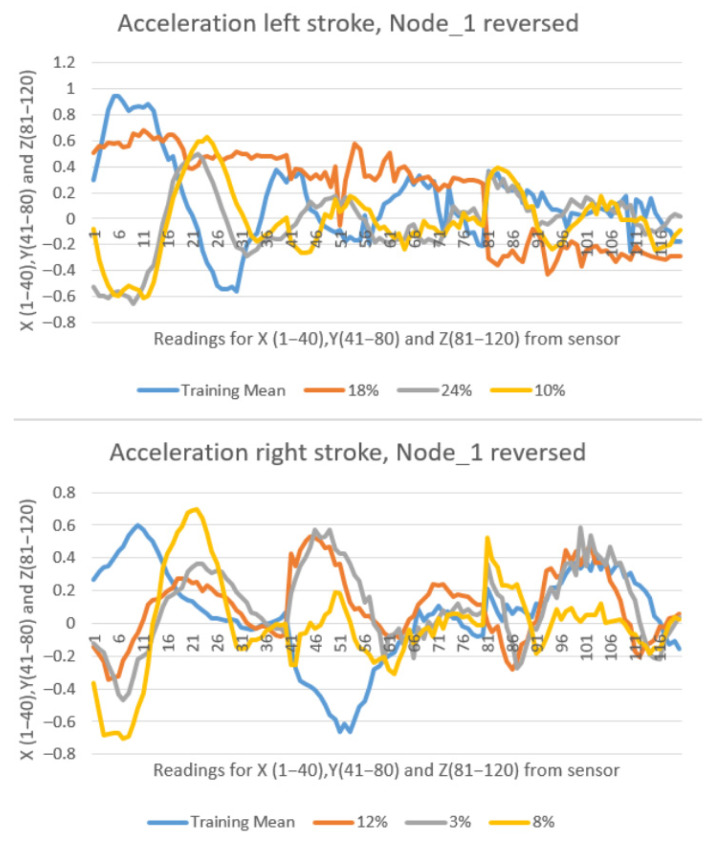
Results with Node_1 rotated from its designated position.

A negative control test was conducted under indoor conditions to evaluate the system’s response when slave nodes were rotated by 180° from their designated positions, as illustrated in Figure 1. Figure 16 presents the output from Node_1, showing the recorded values (orange, gray, and yellow lines) in comparison with the training pattern (blue line). The percentages shown represent the similarity scores calculated for each movement. The results clearly demonstrate that altering node orientation from the trained configuration leads to substantially reduced similarity percentages, thereby preventing the system from operating as intended, even when the movements were executed in accordance with the reference pattern (orange, gray, and yellow lines).

In terms of battery efficiency, all slave nodes were fully charged prior to testing and demonstrated sufficient power to operate for over 3 h of continuous use. During this period, the nodes functioned under various operational states, including calibration, training, voting, and long-distance communication from the master node. After 3 h of active use, all nodes remained powered on for an additional hour in standby mode, with no active tasks being performed.

These results indicate a total runtime of approximately 3 h under load and 1 h in standby. Based on this performance, it can be assumed that the system is capable of supporting at least three separate 1 h sessions on a single battery charge. This duration aligns well with typical SUP rental periods, which often last around 1 h, making the system suitable for integration into commercial rental scenarios.

**Figure 17:**
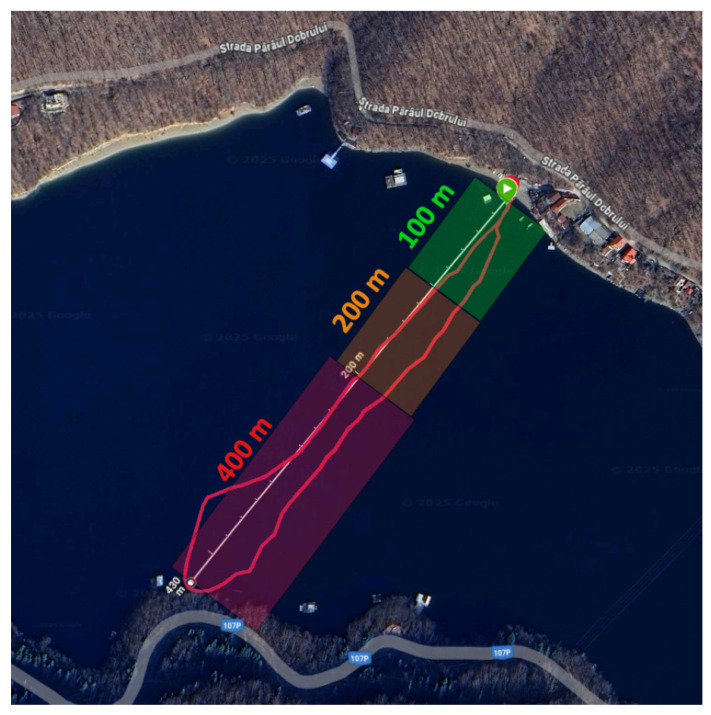
Path of the third test using a smartwatch for GPS monitorization.

A third experiment was made in order to evaluate how far the slave nodes still transmit to the master node using the ESP-NOW Wi-Fi communication protocol. Subject B was on the paddle board, while Subject A was on the lakeshore for monitorization. It should be noted that in this experiment, the subjects switched places. Based on the first and third experiment, in Figure 17, the areas where the system transmitted better and worse can be observed.

During the final experiment, the communication range between the master and slave nodes was evaluated under varying distances. The entire session lasted 20 min, utilizing only the voting phase with previously collected training data.

In the green zone (0–100 m from the master node), communication with the slave nodes was stable. The master device received messages continuously from all IMU-equipped nodes, with no significant loss or corruption of data.

In the orange zone (100–200 m), communication became intermittent. The number of messages received decreased progressively and occasional data anomalies—such as missing or corrupted values—were observed on the web interface.

Beyond 200 m, in the red zone, message reception was sporadic and unreliable. While some transmissions still reached the master node, the content often included unexpected or malformed values, indicating potential data loss or synchronization issues at extended range.

These findings underscore the practical communication limits of the system when using ESP-NOW over open water, highlighting the importance of staying within an effective range—ideally within 100 m—for reliable data collection. This finding is consistent with previous studies, which report comparable communication ranges [27,28,29].

Data collection capabilities were also assessed during this phase by maintaining the voting routine in the active state for a continuous duration of 20 min. Throughout this session, the web interface successfully received and listed a total of 1590 datasets. Each dataset contained 120 sensor readings, along with 2 additional values representing stroke direction and similarity. In total, 193,980 values were transmitted and visualized in real time through the web interface. This result demonstrates the reliability and scalability of the implemented data visualization system. Despite the simplicity of the local web server architecture, it proved capable of handling and displaying a high volume of real-time sensor data, making it a viable tool for field testing and performance monitoring in aquatic sports environments.

In addition, at certain moments in the above-mentioned experiments, there were situations where small boats passed nearby, turns were made, or even an unintentional fall from the SUP. In those cases, the system continued to read, evaluate the movements, and send information back to the master device at the lakeshore. The similarity scores decreased in response to small disturbances caused by other boats.

**Figure 18:**
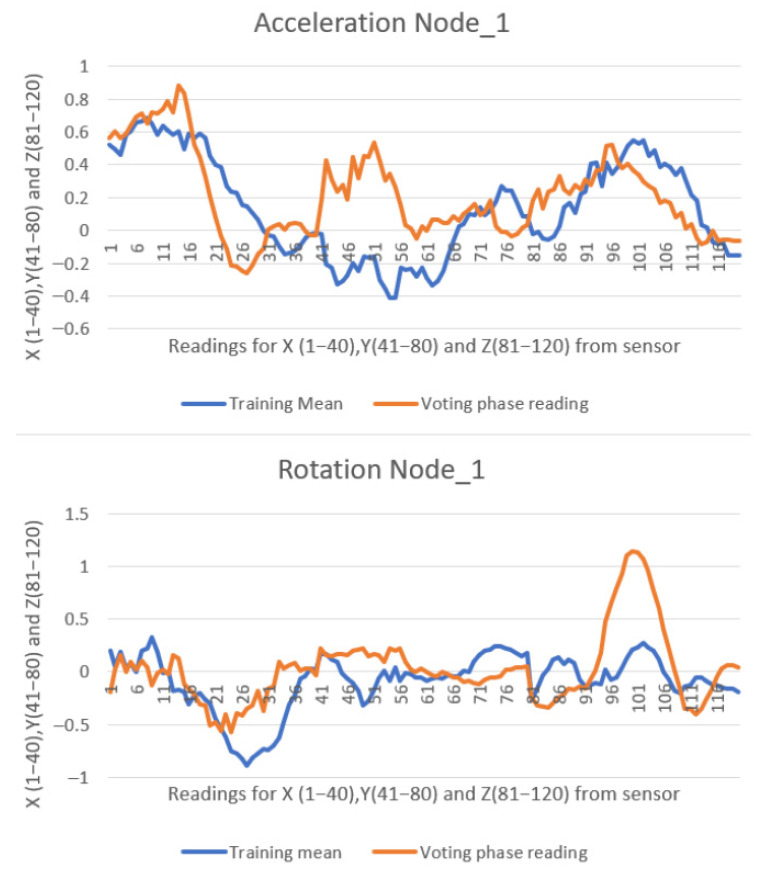
Fifty-nine percent similarity on Node_1 performing a left stroke.

Wind was not the cause of these issues because the weather was calm, but waves were occasionally generated by passing boats. Regarding the architecture of the system, the similarity feedback was only slightly affected, decreasing the similarity output by 10–25%. Figure 18 presents data from Node 1, showing the detection of a left stroke with 59% similarity to the trained pattern, which was influenced by waves generated by passing boats.

**Figure 19:**
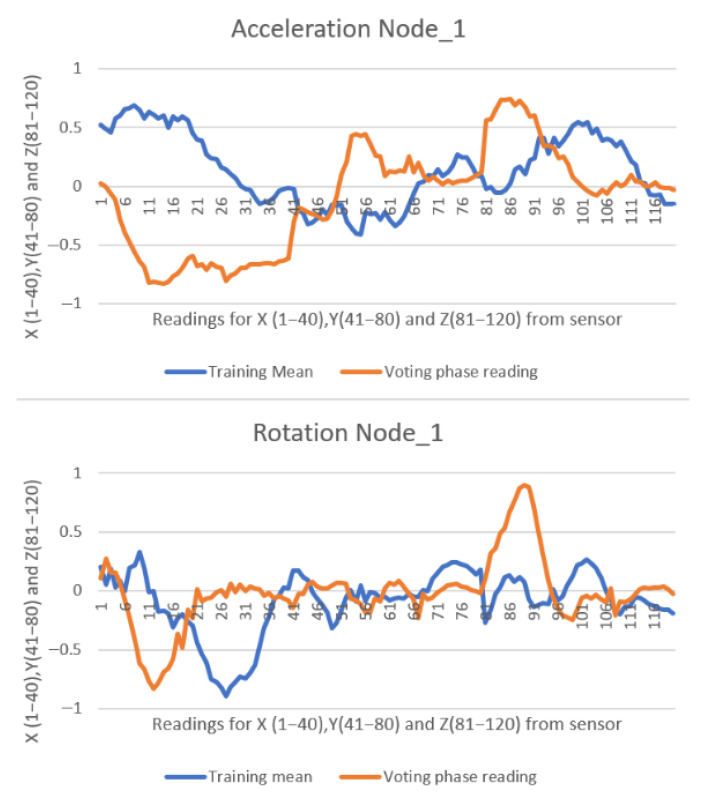
Nine percent similarity on Node_1 performing a left stroke.

In case of a repositioning on the board, or moving the paddle from one hand to another, there was a significant increase in deviation, resulting in a similarity percentage of 0–20%. Some similarity calculations were lost when moving the paddle from one side to the other, whereby the user waited briefly to allow the system to engage autocalibration. Figure 19 presents data from Node 1, showing the detection of a left stroke with 9% similarity to the trained pattern. This was due to moving the paddle from the left hand to the right hand.

## 4. Discussion

The first experiment compared stroke detection using the smartwatch, the second evaluated stroke identification through Dynamic Time Warping (DTW), and the final experiment assessed data transmission performance. Two participants took part in the study. In the first two experiments, Subject A was on the paddleboard, while Subject B was on the lakeshore, whereas in the final experiment, their roles were reversed. In all cases, measurements and monitoring procedures were performed identically; however, each experiment addressed different aspects of the system to provide a comprehensive understanding of its capabilities.

The proposed distributed sensing system, tested on a stand-up paddleboard (SUP) platform, demonstrates promising applications in sports performance monitoring, rehabilitation, and remote coaching. The real-time feedback capabilities and web-based visualization offer immediate insight into stroke patterns and user technique, which is valuable for athletes, therapists, or coaches who cannot be physically present. With slight adaptation, this system could be applied in clinical rehabilitation programs, allowing clinicians to remotely monitor patients’ motor patterns during aquatic therapy.

Furthermore, the system’s capability to store and analyze temporal and directional data enables longitudinal monitoring, which is potentially useful in training regimens or efficiency assessment, over time.

Despite its demonstrated reliability in short-range communication (within a range of 100 m), the system is currently constrained by the limitations of ESP-NOW-based Wi-Fi communication, particularly over water surfaces and at greater distances. The loss of messages in the 100–200 m range and severe degradation beyond that should be taken into account when defining the operational boundaries of the setup.

Another key limitation is the absence of global positioning data (GPS). While GPS integration could offer valuable insights into the correlation between stroke technique and the path traveled, it would increase energy consumption and hardware complexity, thus reducing the current system’s efficient battery performance (over 3 h of active use). Therefore, GPS integration may only be feasible in future revisions, where stroke efficiency analysis relative to course and direction becomes a priority.

Additionally, the system currently relies on data from six IMU-equipped slave nodes, which provide sufficient stroke identification. However, adding two additional sensors—one directly on the paddle and another on the SUP—could enrich the dataset and improve classification accuracy by capturing finer movement nuances, as well as reducing ambiguity, during atypical motion patterns.

A primary constraint encountered during the study was the limited number of available participants and paddleboards for concurrent testing. Although the core objective is to enable the scalable, real-time monitoring of multiple SUP users across a lake, the cost and availability of paddleboards made it difficult to recruit multiple simultaneous participants. To overcome this, future experiments should aim to engage local sports clubs or organize structured group sessions to evaluate the system under realistic, multi-user conditions.

On the technical side, the system’s architecture has potential for scalability and enhanced robustness. The ESP-NOW protocol, chosen for its low-latency and energy-efficient communication, can be extended by multiplexing MAC addresses, thereby allowing additional slave nodes to be incorporated into the network [30]. This approach supports distributed sensing configurations, such as tracking multiple athletes or collecting redundant sensor data from different body parts. Furthermore, the ESP32’s ability to measure Received Signal Strength Indication (RSSI) from up to 6–8 peer devices can serve as a foundation for proximity estimation or relative positioning, adding a spatial layer to performance analytics [31].

To improve the temporal alignment and cross-node correlation of events, future versions of the system should integrate timestamp synchronization. Precise time markers across all nodes will allow for the improved fusion and comparison of data streams, especially when monitoring multiple athletes or paddle segments simultaneously. This enhancement is essential for accurately distinguishing between overlapping strokes or coordinating input from spatially distributed sensors.

In this context, the aim was to evaluate the system in alternative scenarios and environments, such as paddleboarding on a lake. The voting mechanism independently computes, for each node, the similarity percentage between a detected movement and a known reference pattern. This can allow the system to provide direct feedback to the user when a movement is not executed correctly by incorporating vibration motors [11]. After each movement—in this case, strokes—the information is transmitted to the master device, enabling a coach or lifeguard to monitor the user in real time. While timestamping is beneficial for identifying movements on the master device, it is not necessary on the slave devices, given the current system design. The slave devices do not communicate with each other for the following three main reasons: (1) to avoid communication issues if a device is submerged in water, as the system is intended for water sports and a communication dependency is risky in terms of provided output; (2) to prevent excessive computational load that could delay movement feedback; and (3) to further preserve battery life.

Additionally, there is an opportunity to implement adaptive communication protocols. While ESP-NOW remains efficient for short-range, low-latency data exchange, future iterations could dynamically switch to longer-range protocols, such as LoRa or mesh networking, when greater distance or inter-node resilience is required [32,33]. Such hybrid communication strategies would improve the system’s adaptability in larger bodies of water or when operating in sparse network conditions.

From a usability perspective, the current system interfaces with a laptop-hosted web application for real-time data visualization and control. While effective in a controlled test environment, this setup limits mobility and accessibility. The development of mobile or cloud-based interfaces would provide greater flexibility, allowing coaches, athletes, or researchers to remotely access performance metrics via smartphones or tablets. This would also facilitate centralized data logging and post-session analysis without requiring proximity to the data collection device.

In summary, while the present system demonstrates reliable single-user monitoring, its full potential lies in evolving toward a robust, networked solution that is capable of supporting multi-user analysis in open-water settings. Future developments should emphasize expanded networking capabilities, precise timestamping, adaptive communication modes, and cloud-integrated interfaces to support broader deployment in sports science, coaching, and safety applications.

## 5. Conclusions

The research successfully demonstrates the feasibility of a distributed, low-power wireless sensor network for stroke detection and monitoring on a stand-up paddleboard. Through a combination of DTW-based pattern recognition, real-time data visualization, and compact wireless communication using ESP-NOW, the system maintained high detection rates, with an average stroke recognition accuracy of 84.40% across all nodes.

Testing showed stable communication and performance within 100 m from the shore, but message reliability declined significantly beyond this range. A simple web interface efficiently handled high data throughput, processing over 193,000 sensor values in 20 min, proving the system’s robustness for real-world use.

While current limitations, such as short-range Wi-Fi and a lack of GPS, restrict the system’s applicability in certain scenarios, its modularity and low energy requirements make it well-suited for short-distance sports monitoring, rehabilitation, and coaching use-cases. Future improvements, including enhanced synchronization, onboard GPS, and sensor augmentation, could broaden its application and enable a more precise analysis of stroke dynamics and performance outcomes.

Compared to existing commercial solutions, which often focus on single-user, high-cost, limited-scalability designs, the proposed system offers a low-cost, flexible, and extensible platform tailored for multi-user environments in natural water settings. While many market devices provide valuable stroke rate and GPS data, they typically lack the capability for the real-time, networked monitoring of multiple athletes or adaptability to harsh environmental noise conditions.

This study advances the field by introducing a wireless sensor network with synchronized data collection, based on ESP-NOW, which is capable of robust stroke classification despite environmental challenges such as small waves and user instability on the SUP. The integration of timestamp synchronization and the potential for adaptive communication protocols represent novel improvements over conventional approaches. Furthermore, the system’s architecture supports expansion to other water sports, such as wing foiling and kite surfing [34], thereby broadening its applicability and impact.

## Figures and Tables

**Table 1:** Comparison between similar available systems.

System	Price	Metrics	Battery	Usability Highlights
Power Meter	USD 750	Power stroke efficiency	Rechargeable (10–12 h in use)	Robust paddle mounting; no app dependency
Nelo Motion Sensor	USD 50	Stroke angle, force, roll	Rechargeable (several sessions)	Rich data; setup via clamps
SpeedCoach SUP 2	USD 471.91	Speed, rate, DPS, calories	Rechargeable (6–8 h in use)	Waterproof display; SUP-specific layout
Vaaka Cadence Sensor	USD 225	Stroke rate, DPS	AAA batteries (300 h in use)	Lightweight; universal mounting; long battery
Motionize (rowing)	USD 169.99	Stroke timing, rhythm	Rechargeable (several sessions)	Rowing-focused; boat integration

**Table 2:** Second test for stroke identification based on left and right stroke patterns.

Sensor	Total Strokes	Left Strokes	Right Strokes	Total Percentage of Identification/Node
Node 1	40	18	22	85.11%
Node 2	43	16	27	91.49%
Node 3	34	20	14	72.34%
Node 4	41	19	22	87.23%
Node 5	39	18	21	82.98%
Node 6	41	19	22	87.23%

## Data Availability

Data are contained within the article.

## Supplementary Materials

The following supporting information can be downloaded at: https://www.mdpi.com/article/10.3390/s25175307/s1, Video S1: Demonstration of a training session for left and right strokes, including the voting phase performed on the lake.
